# Supplementary material for: Differential Expression of RSK4 Transcript Isoforms in Cancer and Its Clinical Relevance
Source: Int J Mol Sci. 2022 Nov 23;23(23):14569. doi: 10.3390/ijms232314569 (PMC9737342; doi:10.3390/ijms232314569)

Supp Table S1: Matching of GTEx and TCGA tissue samples

GTEX_Tissue	TCGA_Cancer types	Abbreviations	GTEX	TCGA
Bladder	Bladder Urothelial Carcinoma	BLCA	9	407
Breast	Breast Invasive Carcinoma	BRCA	179	1099
Cervix & Uterus	Cervical & Endocervical Cancer<br>Uterine Carcinosarcoma	CESC<br>UCS	88	363
Brain	Glioblastoma Multiforme<br>Brain Lower Grade Glioma	GBM<br>LGG	1141	689
Fallopian Tube	Ovarian Serous Cystadenocarcinoma	OV	5	427
Lung	Lung Adenocarcinoma<br>Lung Squamous Cell Carcinoma	LUAD<br>LUSC	288	1013
Prostate	Prostate Adenocarcinoma	PRAD	100	496
Testis	Testicular Germ Cell Tumor	TGCT	165	154
Esophagus	Esophageal Carcinoma	ESCA	653	182
Pancreas	Pancreatic Adenocarcinoma	PAAD	167	179
Kidney	Kidney Papillary Cell Carcinoma<br>Kidney Clear Cell Carcinoma<br>Kidney Chromophobe	KIRP<br>KIRC<br>KICH	28	886
Liver	Liver Hepatocellular Carcinoma	LIHC	110	371
Muscle	Sarcoma	SARC	1189	349
Colon	Colon Adenocarcinoma	COAD	308	290
Stomach	Stomach Adenocarcinoma	STAD	174	414
Skin	Skin Cutaneous Melanoma	SKCM	556	469
Thyroid	Thyroid Carcinoma	THCA	279	512
Blood	Acute Myeloid Leukemia<br>Diffuse Large B-Cell Lymphoma	AML<br>DLBC	337	220
Adrenal Gland	Adrenocortical Cancer<br>Pheochromocytoma & Paraganglioma	ACC<br>PCPG	128	259

Supp Table S2: Outcomes of univariate and multivariate Cox proportional hazards ratio analysis for LGG, STAD, KIRC, CESC.

Brain Lower Grade Glioma (LGG)				
Variables	Univariate analysis		Multivariate analysis	
	HR(95% CI)	adj-p-value	HR(95% CI)	adj-p-value
RSK4 isoform 1	0.77 (0.71-0.84)	1.64e-08 ***	0.763 (0.689-0.844)	1.49e-07 ***
Family history (Yes/No)	1.23 (0.84-1.79)	0.283		
Gender (male vs female)	1.12 (0.79-1.58)	0.538		
Age (>60 vs ≤ 60)	5.02 (3.28-7.66)	8.3e-14 ***	4.91 (3.19-7.58)	5.75e-13 ***
Headache (Yes/No)	0.859 (0.59-1.25)	0.427		
Recurrence (Yes/No)	2.71 (1.86-3.96)	2.28e-07 ***	2.61 (1.78-3.80)	6.87e-07 ***
**Stomach Adenocarcinoma (STAD)**				
Variables	Univariate analysis		Multivariate analysis	
	HR(95% CI)	adj-p-value	HR(95% CI)	adj-p-value
RSK4 isoform 1	1.08 (1.02-1.15)	1.89e-02 *	1.10 (1.03-1.18)	0.00339 **
Gender (male vs female)	1.19 (0.849-1.67)	0.312		
Age (>60 vs ≤ 60)	1.66 (1.16-2.57)	0.00582 **	2.12 (1.47-3.07)	6.67e-05 ***
Stage.high (I&II vs III&IV)	1.85 (1.33-2.57)	0.000234 ***	1.60 (1.14-2.25)	0.00692 **
Recurrence (Yes/No)	2.50 (1.82-3.44)	1.35e-08 ***	2.33 (1.69-3.20)	1.89e-07 ***
Metastasis (Yes/No)	2.32 (1.36-3.96)	0.00198 **	2.31 (1.32-4.02)	0.00322 **
**Kidney Clear Cell Carcinoma (KIRC)**				
Variables	Univariate analysis		Multivariate analysis	
	HR(95% CI)	adj-p-value	HR(95% CI)	adj-p-value
RSK4 isoform 2	0.93 (0.90-0.97)	6.81e-04 ***	0.943 (0.906-0.981)	0.0038 **
Gender (male vs female)	0.949 (0.696-1.29)	0.742		
Age (>60 vs ≤ 60)	1.83 (1.34-2.49)	0.000128 ***	1.67 (1.22-2.27)	0.0012 **
Stage.high (I&II vs III&IV)	3.74 (2.73-5.13)	< 2e-16 ***	2.59 (1.78-3.76)	6.40e-07 ***
Recurrence (Yes/No)	0.298 (0.110-0.805)	0.017 *	0.168 (0.0617-0.458)	0.0005 ***
Metastasis (Yes/No)	4.32 (3.16-5.90)	< 2e-16 ***	2.62 (1.81-3.79)	3.48e-07 ***
**Cervical & Endocervical Cancer (CESC)**				
Variables	Univariate analysis		Multivariate analysis	
	HR(95% CI)	adj-p-value	HR(95% CI)	adj-p-value
RSK4 isoform 2	1.23 (1.14-1.33)	2.26e-07 ***	1.21 (1.12-1.32)	5.46e-06 ***
Age (>60 vs ≤ 60)	1.83 (0.545-1.10)	0.0196 *	1.29 (0.747-2.21)	0.365
Stage.high (I&II vs III&IV)	2.41 (1.49-3.93)	0.000371 ***	1.93 (1.16-3.20)	0.0111 *
Recurrence (Yes/No)	4.70 (2.95-7.49)	6.77e-11 ***	4.39 (2.74-7.03)	7.33e-10 ***
Metastasis (Yes/No)	2.28 (0.83-6.29)	0.11		

Supp Fig S1: Kruskal Wallis tests – comparison of isoform 1 and 2 expression among both normal and tumour samples

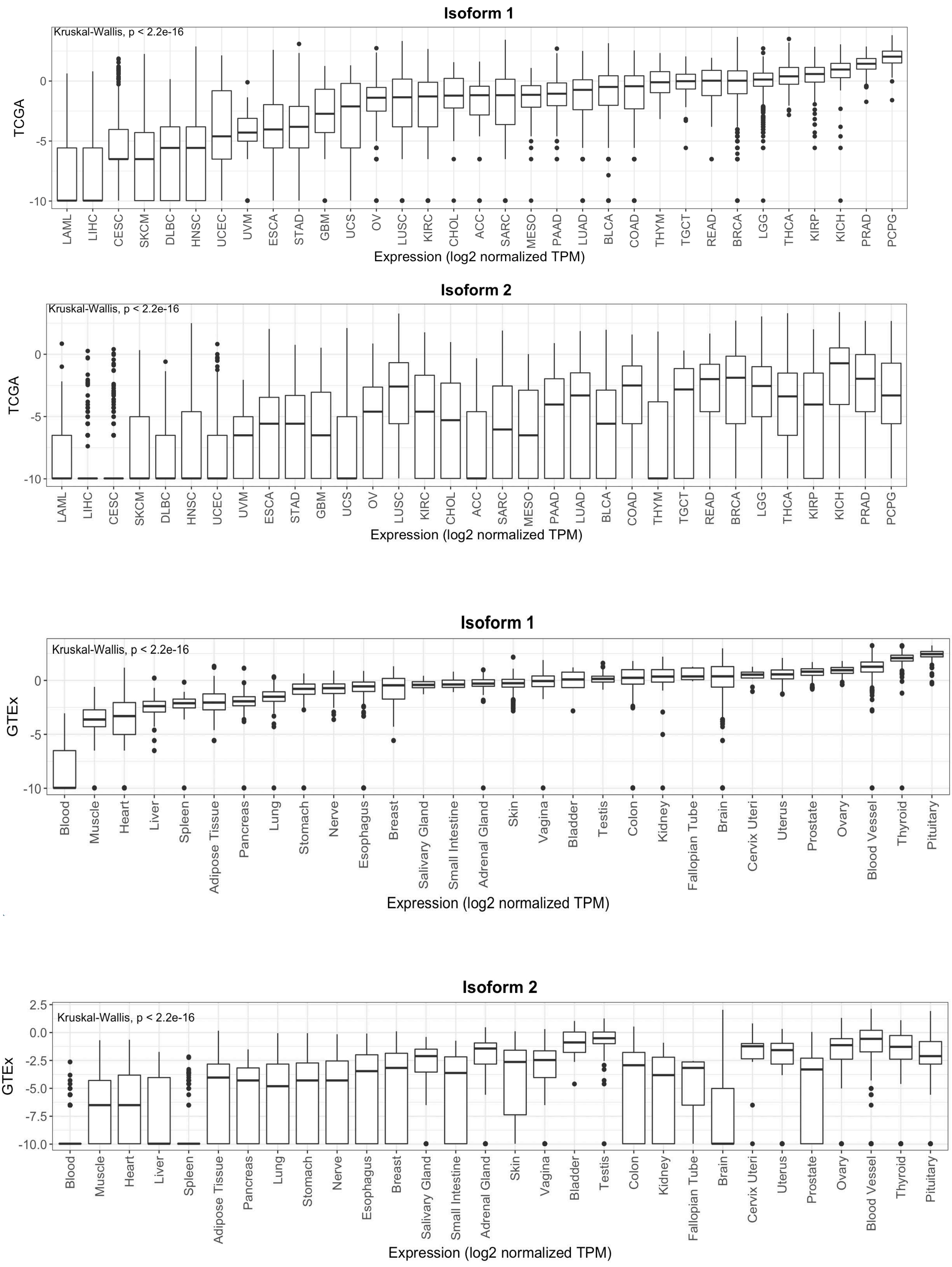

Supp Fig S2: Proportion of infiltrating CAFs and CD4⁺ T cells and overall survival of patients with LGG, KIRK and CESC.

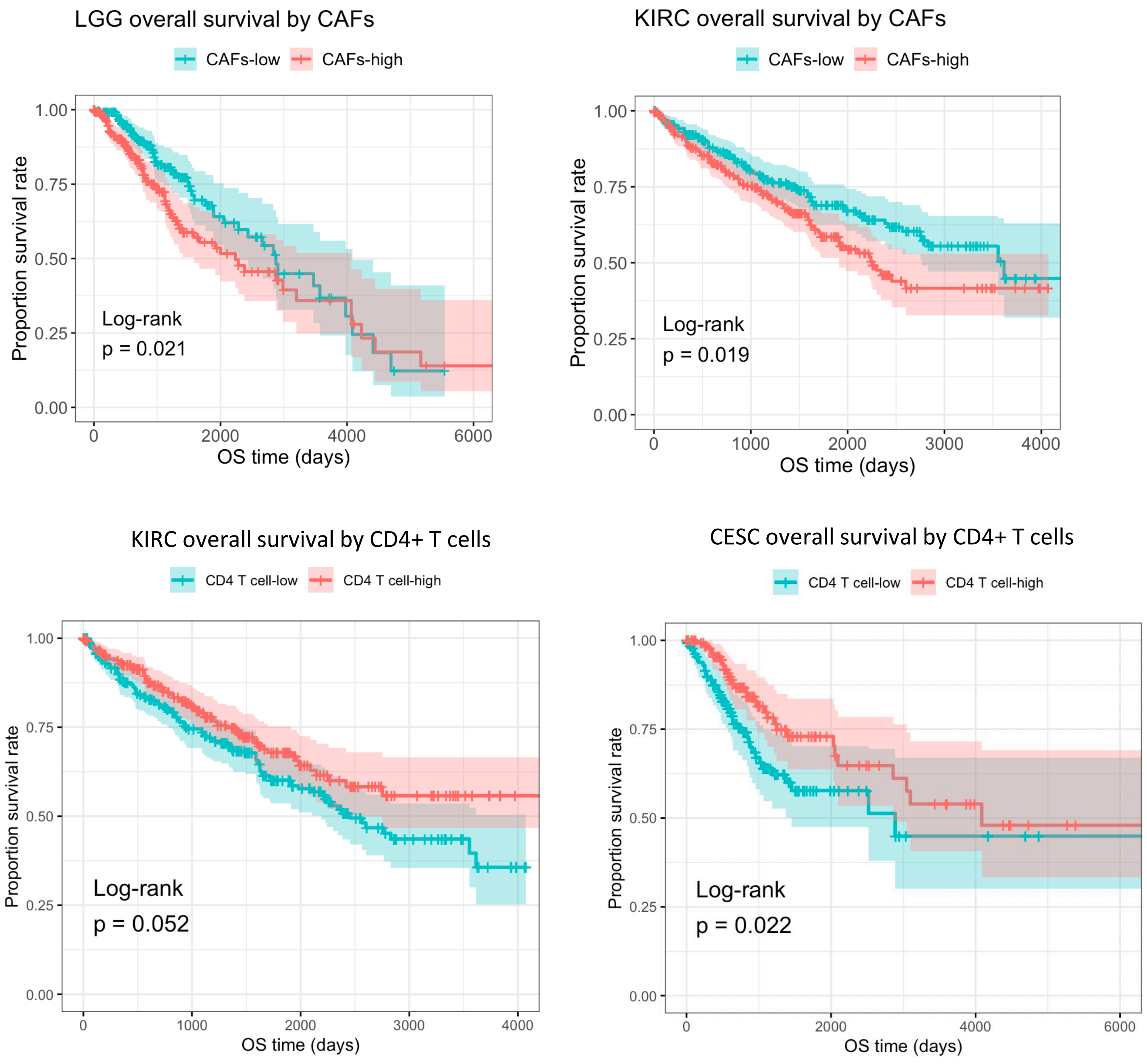

Supplement: Supplementary file 1 [file ijms-23-14569-s001.zip › ijms-2005143-supplementary.pdf]
